# Impacts of Nontuberculous Mycobacteria Isolates in Non-cystic Fibrosis Bronchiectasis: A 16-Year Cohort Study in Taiwan

**DOI:** 10.3389/fmicb.2022.868435

**Published:** 2022-04-18

**Authors:** Chun-Yu Lin, Hung-Yu Huang, Meng-Heng Hsieh, Yueh-Fu Fang, Yu-Lun Lo, Shu-Min Lin, Yu-Tung Huang, Chih-Hsin Yeh, Chun-Hua Wang, Horng-Chyuan Lin

**Affiliations:** ^1^Department of Thoracic Medicine, Linkou Chang Gung Memorial Hospital, Taoyuan, Taiwan; ^2^College of Medicine, Chang Gung University, Taoyuan, Taiwan; ^3^Center for Big Data Analytics and Statistics, Linkou Chang Gung Memorial Hospital, Taoyuan, Taiwan; ^4^Department of Respiratory Therapy, Linkou Chang Gung Memorial Hospital, Taoyuan, Taiwan

**Keywords:** nontuberculous mycobacteria, *Pseudomonas aeruginosa*, bronchiectasis, fungi, non-cystic fibrosis bronchiectasis

## Abstract

**Background:**

The prevalence of nontuberculous mycobacteria (NTM) in patients with chronic respiratory disease has increased. The implication of NTM in non-*CF* bronchiectasis remained controversial. This study investigated the impact of NTM in non-*CF* bronchiectasis in Taiwan.

**Methods:**

Clinical manifestation, imaging, and microbiological data were retrieved from the Chang Gung Research Database, the largest electronic medical record-based database in Taiwan. Patients with bronchiectasis during 2001–2016 were included. Cox proportional hazard model was employed to compare outcomes between patients with negative and positive NTM isolates after 1:1 propensity score matching.

**Results:**

A total of 19,647 non-*CF* bronchiectasis patients were enrolled and 11,492 patients were eligible for analysis after exclusion screening. Finally, patients with negative and positive NTM isolates—650 each—were analyzed after propensity score matching. The patients with negative NTM isolates were divided into three groups: *Pseudomonas aeruginosa* isolates (*n* = 53); fungus isolates (*n* = 26); and concomitant *P. aeruginosa* and fungus isolates (*n* = 8). The patients with positive NTM isolates were divided into five groups: single NTM isolate (*n* = 458); multiple NTM isolates (*n* = 60); concomitant NTM and *P. aeruginosa* isolates (*n* = 89); concomitant NTM and fungus isolates (*n* = 33); and concomitant NTM, *P. aeruginosa*, and fungus isolates (*n* = 10). Patients with *P. aeruginosa* isolates; concomitant NTM and *P. aeruginosa* isolates; concomitant NTM, *P. aeruginosa*, and fungus isolates had independently associated with respiratory failure and death. Patients with single or multiple NTM isolates were not related to ventilator use, but both were independent risk factor for mortality.

**Conclusion:**

NTM, either combined with *P. aeruginosa* or fungus, exhibited more frequent exacerbations in non-*CF* bronchiectasis patients. Moreover, NTM predicted mortality in non-*CF* bronchiectasis patients and were also correlated to respiratory failure while concomitantly isolated with *P. aeruginosa* and fungus.

## Introduction

Bronchiectasis is a chronic lung disease with heterogeneous clinical features and outcomes. It is characterized by irreversible dilatation of the bronchi with airway remodeling due to recurrent airway infection, inflammation, and impaired mucociliary clearance. These structural changes in the lung lead to further infections and disease-associated exacerbations, resulting in a vicious circle (Hsieh et al., 2013a,b; Richardson et al., 2019). Recently, increasing evidence has revealed a low-diversity microbiome in severe bronchiectasis and its association with rapid lung function decline (Rogers et al., 2013; Richardson et al., 2019; Woo et al., 2019).

*Pseudomonas aeruginosa* is the most common pathogen in bronchiectasis, driving airway neutrophil-mediated inflammation, and is associated with increased pulmonary function decline and the risks of exacerbation, hospital admission, and mortality (Martinez-Garcia et al., 2007; Finch et al., 2015; Richardson et al., 2019; Menendez et al., 2020).

Hence, chronic colonization by *P. aeruginosa* is a crucial component in severity scoring systems, including the Bronchiectasis Severity Index (BSI) and FACED score (Chalmers et al., 2014; Martinez-Garcia et al., 2014).

Nontuberculous mycobacteria (NTM) is often associated with bronchiectasis, but the role of NTM in bronchiectasis is unclear (Bonaiti et al., 2015; Richardson et al., 2019). Elderly women with a low body mass index have been found to be susceptible to NTM isolates in non-cystic fibrosis (*CF*) bronchiectasis (Aksamit et al., 2017; Lipman et al., 2020). Recently, Faverio et al. (2016) revealed that patients with pulmonary NTM disease are more likely to experience body weight loss and have a lower number of pulmonary exacerbations than patients with chronic *P. aeruginosa* infection (Faverio et al., 2016). Furthermore, Wagner et al. (2020) demonstrated that if physicians do not manage NTM-related lung disease, this neglect may significantly increase the risk of mortality (Wagner et al., 2020). Nevertheless, in a Korean study, Kwak et al. (2020) reported that new-onset NTM pulmonary disease in bronchiectasis worsened radiographic lesions but did not change the BSI (Kwak et al., 2020).

*Aspergillus* is a major fungal genus in bronchiectasis and may be correlated with poor lung function (Mac Aogain et al., 2018; Maiz et al., 2018). Chronic antibiotic treatment may be one of the most significant risk factors for positive fungal isolation (Maiz et al., 2015, 2018). However, the role of fungal isolates in bronchiectasis, except for causing invasive infections, is unclear. In our previous report, patients with positive NTM isolates, positive *P. aeruginosa* isolates, and concomitant NTM and *P. aeruginosa* isolates exhibited high BSI scores and frequent exacerbations and emergency department (ED) visits (Hsieh et al., 2018c).

In the current study, we investigated the clinical implications of concomitant NTM, *P. aeruginosa*, and fungal isolates in patients with non-*CF* bronchiectasis. The results of this study add to the knowledge of NTM in bronchiectasis.

## Materials and Methods

### Data Source

In this study, we used the Chang Gung Research Database (CGRD) to construct a multicenter bronchiectasis cohort for the period of 2001–2016. The CGRD is an electronic medical record database of Chang Gung Medical Foundation, which is the largest hospital system in Taiwan, comprising three medical centers (Linkou, Taipei, and Kaohsiung branches) and four regional hospitals (Taoyuan, Keelung, Chiayi, and Yunlin branches).^1^ These hospitals are located across Taiwan. The data in the CGRD include demographic data, inpatient and outpatient records, diagnostic codes, medications, and reports of microbiological, imaging study, and functional examination data (Shao et al., 2019; Huang et al., 2020).

### Bronchiectasis Cohort

We searched the electronic medical records in the CGRD between 1 January 2001, and 31 December 2016, and retrieved data of patients with a diagnosis of bronchiectasis. The bronchiectasis diagnosis was recorded according to the *International Classification of Diseases Clinical Modification* code 494.0 or 494.1 (ninth revision) or J47 (tenth revision). The diagnosis of bronchiectasis was confirmed by the interpretation of chest radiography or high-resolution computed tomography (HRCT), which was independently reviewed by a radiologist (Nicotra et al., 1995; Amaral et al., 2015). Patients with at least two primary bronchiectasis diagnoses on different dates during outpatient visits or one diagnosis during hospitalization were included in the cohort. The index date was defined as the date of the first bronchiectasis diagnosis. The cohort only included patients aged ≥20 years. Demographic data were collected. Patients who met the following criteria were excluded as: previous diagnosis of pulmonary tuberculosis, sputum not evaluated for NTM isolation, incomplete medical records, and missed follow-up.

### Microbiology and Exacerbation Definition

We collected sputum microbiology reports within 6 months after the index date. Sputum cultures for bacteria, mycobacteria, and fungus were recorded (Finch et al., 2015). NTM pulmonary disease was diagnosed as recommended by the American Thoracic Society (ATS) and Infectious Diseases Society of America (IDSA; Daley et al., 2020). NTM species were identified in microbiology laboratories through matrix-assisted laser desorption ionization time-of-flight mass spectrometry. “Multiple NTM isolates” was defined as more than two types of NTM infection. Spirometry was performed according to the American Thoracic Society and the European Respiratory Society criteria (Miller et al., 2005).

We defined acute exacerbations (AEs) as an event that was clinically diagnosed by a physician and required an antibiotic prescription for acute onset of increasing cough, worsening dyspnea, and changes in sputum characteristics (e.g., volume, consistency, and purulence). Furthermore, we recorded the frequency of AEs, ED visits, as well as hospitalization; ventilator usage due to bronchiectasis AE; and all-cause mortality (Hsieh et al., 2018c).

### Statistics

A nonbalanced distribution of clinical characteristics between the study patients with negative and positive NTM isolates would have seriously confounded the results. Therefore, we performed propensity score matching to make the two groups comparable. The propensity score was the predicted probability of positive NTM isolates based on logistic regression. Covariates included in the propensity score were variables theoretically and clinically related to outcomes, including demographics (age and sex), pulmonary function, extensions in HRCT reports, etiology, comorbidities, and Bronchiectasis Etiology and Comorbidity Index (BACI) scores at baseline (Table 1). The groups were matched at a 1:1 ratio by using a greedy nearest-neighbor algorithm with a caliper of 0.2.

**Table 1:** Clinical characteristics of non-*CF* bronchiectasis patients in the six groups according to the pathogen isolated from sputum samples.

	Control (*n* = 563)	*P. aeruginosa* (*n* = 53)	Fungus (*n* = 26)	*P. aeruginosa* + Fungus (*n* = 8)	Single NTM (*n* = 458)	Multiple NTM (*n* = 60)	NTM + *P. aeruginosa* (*n* = 89)	NTM + Fungus (*n* = 33)	NTM + *P. aeruginosa* + Fungus (*n* = 10)
Age (mean ± SD)	64.1 ± 14.5	65.4 ± 14.1	67.6 ± 15.2	67 ± 10.1	64.6 ± 13.5	61.1 ± 13.0	66.9 ± 13.8	64.4 ± 12.3	73.6 ± 10.0^*^
Male, *n* (%)	225 (40)	21 (40)	8 (31)	2(25)	177 (39)^*^	20 (33)	37 (42)	16 (48)	6 (60)
Pulmonary function									
FEV1 ≧ 80%	175 (52)	12 (36)	10 (50)	1 (33)	146 (45)	14 (35)	14 (21)^*^	11 (39)	3 (30)
50 ≦ FEV1 < 80%	108 (32)	9 (27)	6 (30)	0 (0)	88 (27)	13 (33)	21 (31)	6 (21)	1 (10)
FEV1 < 50%	62 (18)	13 (39)^*^	3 (15)	2 (67)	41 (13)^*^	5 (13)	26 (38)^*^	8 (29)	5 (50)^*^
HRCT									
Localized, *n* (%)	109 (53)	18 (72)	2 (20)^*^	0 (0)^*^	134 (52)	15 (44)	27 (44)	8 (42)	4 (44)
Bilateral, *n* (%)	49 (24)	4 (16)	5 (50)	4 (100)^*^	62 (24)	10 (29)	20 (32)	3 (16)	4 (44)
Diffuse, *n* (%)	46 (23)	3 (12)	3 (30)	0 (0)	60 (23)	9 (26)	15 (24)	8 (42)^*^	1 (11)
Etiology									
Post-infection	345 (61)	49 (92)^*^	19 (73)	6 (75)	273 (60)	30 (50)^*^	82 (92)^*^	25 (76)	9 (90)
Idiopathic	109 (19)	0 (0)^*^	3 (12)	2 (25)	94 (21)	20 (33)^*^	3 (3)^*^	5 (15)	1 (10)
Comorbidity, *n* (%)									
Ischemic heart disease	66 (12)	6 (11)	2 (8)	0 (0)	32 (7)^*^	8 (13)	16 (18)^*^	2 (6)	1 (10)
Stroke	63 (11)	6 (11)	3 (12)	0 (0)	37 (8)^*^	6 (10)	9 (10)	1 (3)	2 (20)
Diabetes mellitus	71 (13)	7 (13)	2 (8)	3 (38)	58 (13)	7 (12)	12 (13)	5 (15)	1 (10)
Liver disorder	76 (14)	4 (8)	4 (15)	0 (0)	66 (14)	6 (10)	14 (16)	6 (18)	2 (20)
Chronic kidney disease	37 (7)	8 (15)^*^	1 (4)	1 (13)	34 (7)	6 (10)	5 (6)	4 (12)	1 (10)
COPD	204 (36)	20 (38)	8 (31)	5 (63)	149 (33)^*^	21 (35)	41 (46)^*^	8 (24)	6 (60)
Asthma	116 (21)	18 (34)^*^	5 (19)	2 (25)	91 (20)	6 (10)^*^	30 (34)^*^	10 (30)	4 (40)
GERD	54 (10)	5 (9)	2 (8)	1 (13)	35 (8)^*^	1 (2)^*^	9 (10)	7 (21)^*^	0 (0)
Hematological malignancy	158 (28)	12 (23)	9 (35)	1 (13)	142 (31)^*^	13 (22)	26 (29)	8 (24)	4 (40)
Connective tissue disease	11 (2)	1 (2)	0 (0)	0 (0)	53 (12)	6 (10)	14 (16)	3 (9)	2 (20)
Osteoporosis	69 (12)	9 (17)	4 (15)	1 (13)	58 (13)	6 (10)	18 (20)^*^	1 (3)	2 (20)
BACI score	5.4 ± 5.8	5.8 ± 5.7	6.2 ± 6.3	5.8 ± 4.3	5.2 ± 5.5	4.3 ± 4.5^*^	7.2 ± 6.3^*^	5.7 ± 6.4	7.9 ± 5.1
Time from bronchiectasis to NTM isolates, years					3.2 ± 4.3	3.9 ± 4.7	5.3 ± 4.8	3.3 ± 4.0	4.5 ± 5
Followed period, years	3.2 ± 3.7	3.4 ± 3.9	2.2 ± 3.5	1.5 ± 1.8^*^	2.7 ± 1.9^*^	2.5 ± 1.7^*^	2.5 ± 1.8^*^	3.2 ± 2.2^*^	1.7 ± 1.3^*^
Exacerbations									
Hospitalization	1.1 ± 0.3	1.1 ± 0.6	1.2 ± 0.6	2 ± 1.4	1.3 ± 0.7^*^	1.5 ± 0.9^*^	1.7 ± 1.0^*^	1.6 ± 0.7^*^	2 ± 0.8^*^
ED visit	1.6 ± 1.2	1.6 ± 1.5	2 ± 1.3	2 ± 0.1	2.2 ± 1.5^*^	2.3 ± 1.8^*^	2.9 ± 2^*^	3.1 ± 1.7^*^	3.2 ± 1.7^*^
Clinics	2.9 ± 3.3	4.3 ± 5.5	3.8 ± 2.7	1.5 ± 0.7	4.2 ± 4.1^*^	6.5 ± 7.9^*^	5.7 ± 6.2^*^	5.6 ± 4.2^*^	6.3 ± 4.7^*^
Ventilator use (1 y), *n* (%)	60 (11)	31 (58)^*^	7 (27)^*^	4 (50)^*^	42 (9)	4 (7)	34 (38)^*^	5 (15)	7 (70)^*^
Ventilator use (3 y), *n* (%)	93 (17)	33 (62)^*^	9 (35)^*^	5 (63)^*^	71 (16)	8 (13)	44 (49)^*^	5 (15)	7 (70)^*^
Death (1 y), *n* (%)	14 (2)	4 (8)	2 (8)	1 (13)	10 (2)	1 (2)	9 (10)^*^	0 (0)	2 (20)^*^
Death (3 y), *n* (%)	22 (4)	6 (11)^*^	2 (8)	1 (13)	31 (7)^*^	5 (8)	13 (15)^*^	0 (0)	4 (40)^*^

* *p < 0.05 for comparison between the pathogen isolates groups and the control group*.

We used the analysis of variance test and Student’s *t*-test to compare numerical data. The chi-square test was used to compare independent categorical data. For ventilator usage and all-cause mortality, we plotted Kaplan–Meier survival curves. We compared the risk of ventilator usage and all-cause mortality between groups by using a Cox proportional hazards regression model. A *p* < 0.05 was considered statistically significant. Data processing and analyses were performed using SAS Enterprise Guide version 7.1 (SAS Institute, Inc.).

## Results

### Study Population

**Figure 1:**
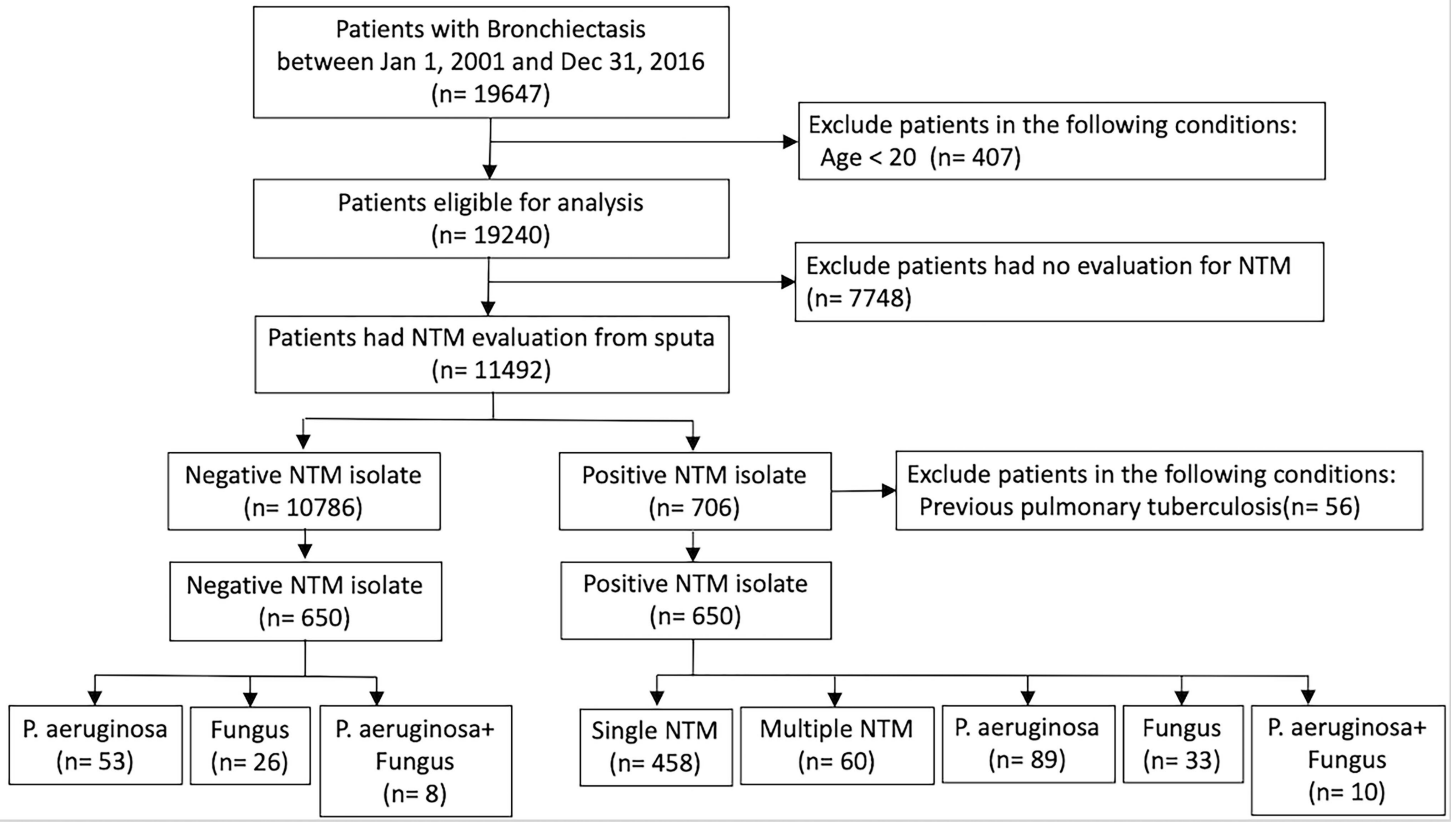
Flowchart of the study design. NTM, nontuberculous mycobacteria.

In total, 19,647 patients with at least two outpatient visits or one inpatient record between 2001 and 2016 in the CGRD were included in our bronchiectasis cohort. After excluding patients <20 years old or with any of the aforementioned clinical exclusion criteria, 11,492 patients remained. Among them, 10,786 patients had negative NTM cultures and 706 patients had positive NTM cultures in their sputum. Additionally, among patients with positive NTM isolates, those with a previous diagnosis of pulmonary tuberculosis were excluded; thus, 650 patients eligible for analysis remained (Figure 1). After propensity score matching, the distributions of baseline characteristics, comorbidities, pulmonary function, and HRCT finding were similar between the negative NTM isolate and positive NTM isolate groups (Supplementary Table S1). The negative NTM group was divided into three groups: *Pseudomonas aeruginosa* isolates; fungus isolates; and concomitant *P. aeruginosa* and fungus isolates. The positive NTM group was further divided into five groups: single NTM isolate; multiple NTM isolates; concomitant NTM and *P. aeruginosa* isolates; concomitant NTM and fungus isolates; and concomitant NTM, *P. aeruginosa*, and fungus isolates. The control group represents patients who had no any pathogen identified from sputum (Figure 1; Table 1).

### Clinical Characteristics

The clinical characteristics of the nine groups according to the pathogen isolated from sputum samples are summarized in Table 1. Patients with concomitant NTM, *P. aeruginosa*, and fungus isolates were older than those in the control group (73.6 ± 10.0 years versus 64.4 ± 14.5 years). Compared with the control group, patients with *P. aeruginosa* isolates, concomitant NTM and *P. aeruginosa* isolates, concomitant NTM, *P. aeruginosa*, and fungus isolates had poorer forced expiratory volume in 1 s (FEV1). Patients who had multiple NTM isolates had the lowest BACI score (4.3 ± 4.5), whereas patients with concomitant NTM and *P. aeruginosa* isolates had significantly higher BACI scores (7.2 ± 6.3) than those in the other groups. Patients with positive NTM isolate groups, either combined with *P. aeruginosa* or fungus, exhibited significantly more frequent exacerbations than those in the control group (including hospitalization, ED visits, and clinic visits; Table 1).

### Mycobacterial Species

In this study, 650 patients were positive for NTM isolates: 458 patients had a single NTM isolate and 60 patients had multiple NTM isolates. The *Mycobacterium avium*–*intracellulare* complex was the most common NTM species found, followed by *M. fortuitum* and *M. abscessus*. NTM species were not significantly different between the groups (Supplementary Table S2).

### Outcomes

**Figure 2:**
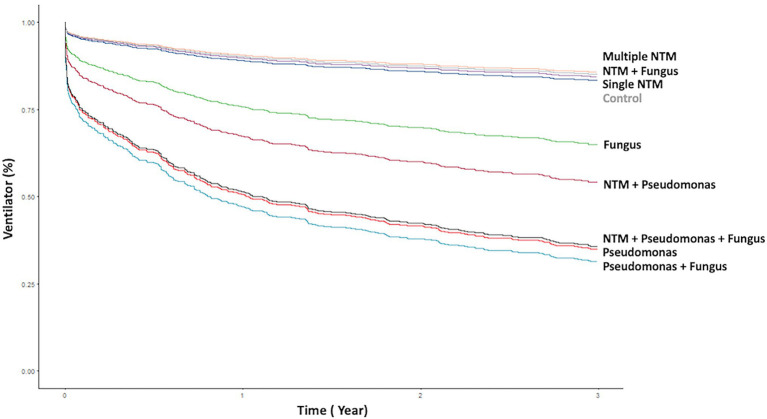
Kaplan–Meier curve of ventilator use according to different pathogen isolates from sputum. NTM, nontuberculous mycobacteria.

**Figure 3:**
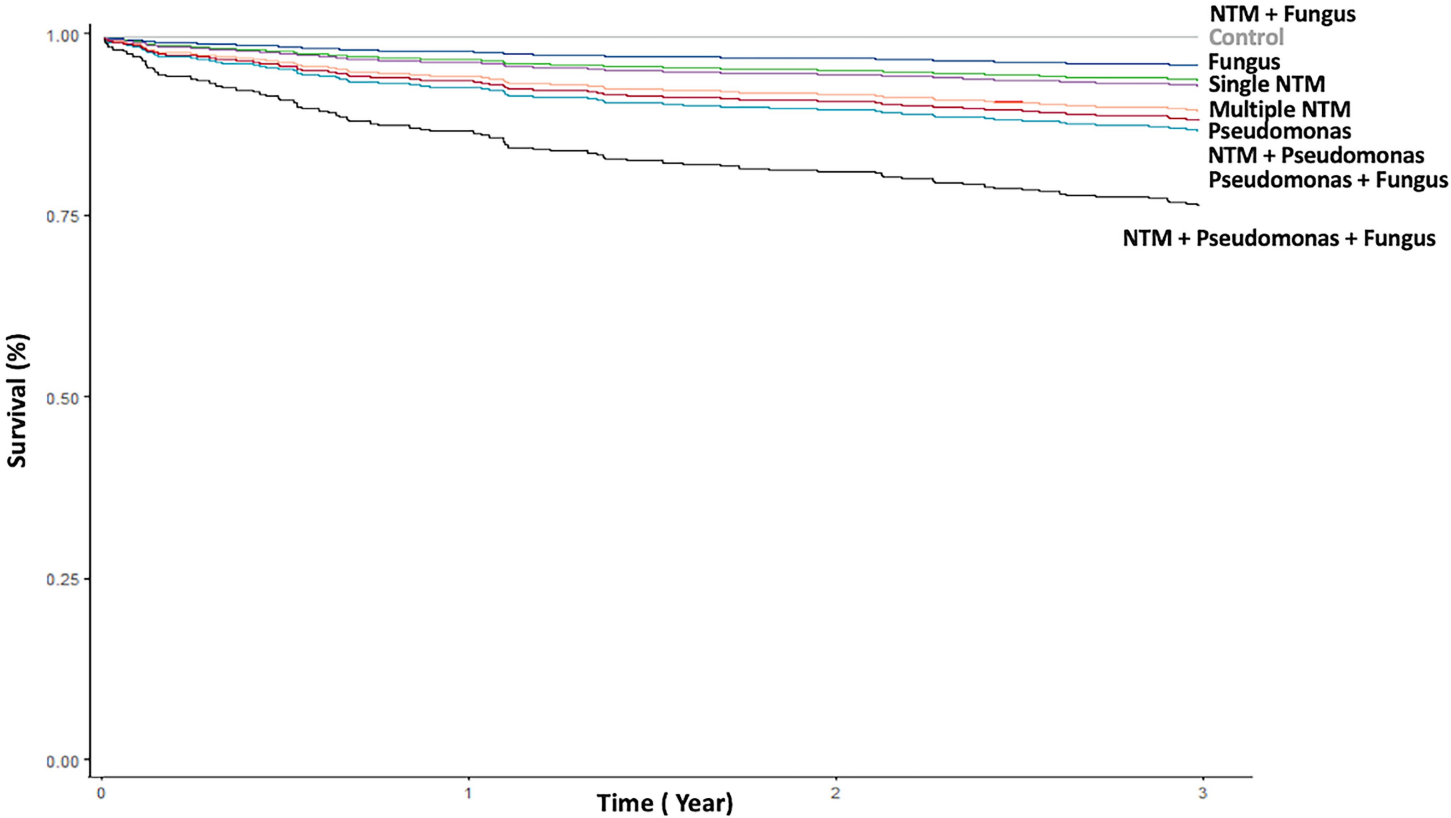
Kaplan–Meier curve of death according to different pathogen isolates from sputum. NTM, nontuberculous mycobacteria.

Patients with *P. aeruginosa* and/or fungus isolates; concomitant NTM, *P. aeruginosa*, and/or fungus isolates had the significantly frequent ventilator use (Table 1, Figures 2, 3). Patients with *P. aeruginosa* and/or fungus isolates, concomitant NTM and *P. aeruginosa* isolates; concomitant NTM, *P. aeruginosa*, and fungus isolates; and high BACI score had independently significantly high risk of respiratory failure (Figure 2; Table 2). Patients with single or multiple NTM isolates were not related to ventilator use, despite the virulence of different NTM species (Table 2; Supplementary Table S3).

**Table 2:** Hazzard ratio of ventilator use according to different pathogen isolates from sputum.

	Univariate			Multivariate		
	HR	95% CI	*P* value	HR	95% CI	*P* value
Age	1.02	1.00–1.03	0.0104	1.01	1.00–1.02	0.1109
BACI score	1.06	1.04–1.09	<0.0001	1.05	1.03–1.07	<0.0001
*P. aeruginosa*	6.12	4.11–9.11	<0.0001	6.01	4.03–8.95	<0.0001
Fungus	2.38	1.20–4.72	0.013	2.41	1.21–4.78	0.0119
*P. aeruginosa* + Fungus	6.19	2.51–15.22	<0.0001	6.51	2.64–16.06	<0.0001
Single NTM	0.91	0.67–1.24	0.5521			
Multiple NTM	0.77	0.37–1.59	0.4807			
NTM + *P. aeruginosa*	3.8	2.65–5.44	<0.0001	3.45	2.41–4.95	<0.0001
NTM + Fungus	0.91	0.37–2.24	0.8155	0.88		
NTM + *P. aeruginosa* + Fungus	7.11	3.29–15.35	<0.0001	5.9	2.72–12.80	<0.0001

NTM, nontuberculous mycobacteria; HR, hazard ratio; and BACI, Bronchiectasis Etiology and Comorbidity Index. ^*^p < 0.05 for comparison between the pathogen isolates groups and the control group.

*P. aeruginosa* isolates; single and multiple NTM isolates; concomitant NTM and *P. aeruginosa* isolates; concomitant NTM, *P. aeruginosa*, and fungus isolates; old age; and high BACI score were independent risk factor for mortality (Figure 3; Table 3). Because patients with concomitant NTM and fungus isolates had no death events, the risk for mortality could not be calculated in this group. With regard to different NTM species, patients had *M. avium–intracellulare complex* isolate or multiple NTM isolates demonstrated worst mortality (Supplementary Table S4).

**Table 3:** Hazzard ratio of mortality according to different pathogen isolates from sputum.

	Univariate			Multivariate		
	HR	95% CI	*P* value	HR	95% CI	*P* value
Age	1.05	1.02–1.07	0.0005	1.05	1.03–1.07	<0.0001
BACI score	1.08	1.04–1.12	<0.0001	1.06	1.03–1.09	0.0001
*P. aeruginosa*	3.02	1.22–7.45	0.0161	2.81	1.14–6.95	0.0248
Fungus	2.06	0.49–8.77	0.3274			
*P. aeruginosa* + Fungus	3.55	0.48–26.32	0.2167			
Single NTM	1.74	1.01–3.01	0.0462	1.79	1.04–3.09	0.0374
Multiple NTM	2.15	0.81–5.67	0.1239	2.82	1.06–7.47	0.0374
NTM + *P. aeruginosa*	4.02	2.03–7.98	0.005	3.21	1.61–6.39	0.0009
NTM + Fungus	0	0	–	0	0	–
NTM + *P. aeruginosa* + Fungus	11.94	4.11–34.65	<0.0001	7.43	2.55–21.69	0.0002

NTM, nontuberculous mycobacteria; HR, hazard ratio; and BACI, Bronchiectasis Etiology and Comorbidity Index. ^*^p < 0.05 for comparison between the pathogen isolates groups and the control group.

## Discussion

The present study is the multicenter observational study to investigate the clinical implications of NTM and other concomitant pathogens identified from sputum samples in patients with non-*CF* bronchiectasis over one-decade period in Taiwan. Patients with positive NTM, either combined with *P. aeruginosa* or fungus isolates, demonstrated significantly more frequent exacerbations. Having *P. aeruginosa* isolates, concomitant NTM and *P. aeruginosa* isolates, concomitant NTM, *P. aeruginosa*, and fungus isolates were associated with significantly poorer pulmonary function. These patients also exhibited independently higher risk of respiratory failure and mortality. More importantly, our results demonstrated that not only polymicrobial isolates patients, who had single or multiple NTM isolates were independent predictor for mortality.

In one meta-analysis published in 2014, the prevalence of NTM in patients with bronchiectasis was 9.3%, and this condition has been mainly reported in Asian populations (Chu et al., 2014; Zhang et al., 2019). The clinical implications of NTM in patients with chronic respiratory disease are controversial. Faverio et al. demonstrated that patients with pulmonary NTM disease may have lower disease severity, lower BSI score, and fewer exacerbations than patients with chronic *Pseudomonas* infection (Faverio et al., 2016). Furthermore, Kwak et al. (2020) reported in a Korean study that NTM pulmonary disease in bronchiectasis worsened radiographic lesions but did not affect the BSI score (Kwak et al., 2020). However, these studies did not analyze patients with concomitant NTM and other pathogen isolates, including *P. aeruginosa* and fungus, which also have clinical implications in bronchiectasis. Huang et al. determined that patients with chronic obstructive pulmonary disease (COPD) who had NTM isolates were more likely to experience exacerbations compared with those without isolates (Huang et al., 2012). Moreover, they found that patients with COPD who had multiple NTM isolates exhibited a greater decline in FEV1 than did those with a single NTM or no isolates (Huang et al., 2012). Patients with bronchiectasis with positive NTM isolates had significant FEV1 declines and frequent annual exacerbations, ED visits, and hospitalizations (Hsieh et al., 2018c). Furthermore, concomitant NTM and *P. aeruginosa* isolates in non-*CF* bronchiectasis was associated with major pulmonary function decline and considerable disease severity (Hsieh et al., 2018c). Because it consisted of a small sample size, we could not further investigate differences in the clinical implications of single and multiple NTM isolates. In current study, 19,647 patients diagnosed with bronchiectasis from 2001 to 2016 were obtained from the CGRD database, and after application of the exclusion criteria, 1,300 patients were enrolled for the analysis. We demonstrated that among patients, having positive NTM isolates, combined with either *P. aeruginosa* or fungus isolates, was positively correlated with frequent exacerbations, including hospitalizations, ED visits, and clinic visits.

Patients had fungus isolates or concomitant *P. aeruginosa* and fungus isolates presented higher risk of respiratory failure. Although patients with single or multiple NTM isolates had no impact on ventilator use, both were strongly related to mortality. Moreover, having *P. aeruginosa* isolates; concomitant NTM and *P. aeruginosa* isolates; and concomitant NTM, *P. aeruginosa*, and fungus isolates were associated with a high risk of ventilator use and mortality. Recently, Huang et al. demonstrated BACI score ≥ 6 had a significantly higher cumulative incidence of respiratory failure and mortality after severe bronchiectasis exacerbations (Huang et al., 2021). We also showed that high BACI score independently predicted respiratory failure and mortality.

NTM causing lots of human diseases, including chronic pulmonary disease, disseminated disease in severely immunocompromised patients, skin, and soft tissue infection, superficial lymphadenitis (To et al., 2020). Pulmonary disease accounts for 80–90% of NTM-associated diseases, the most common species were *Mycobacterium avium complex, Mycobacterium kansasii, and Mycobacterium abscessus* (Johnson and Odell, 2014; To et al., 2020; Zhu et al., 2021). Infection with *Mycobacterium abscessus* may be associated with worse clinical outcomes for the high resistance rate to macrolides. Similar to previously reported studies, *mycobacterium avium–intracellulare complex* was the most common NTM species found in our patients, followed by *M. fortuitum* and *M. abscessus*. There was no NTM species correlated to respiratory failure. But we demonstrated that patients with *Mycobacterium avium complex* or multiple NTM isolates had independently higher mortality.

*P. aeruginosa* is a well-documented pathogen in bronchiectasis, which is associated with increased risk of death, hospital admissions, and exacerbations (Rogers et al., 2014; Finch et al., 2015). Furthermore, chronic colonization by *P. aeruginosa* is a crucial aspect of both BSI and FACED score (Chalmers et al., 2014; Martinez-Garcia et al., 2014). *Aspergillus* spp. is clinically important in severe asthma, COPD, and *CF* (Baxter et al., 2013; Leung et al., 2017; Mac Aogain et al., 2018; Lin et al., 2020). In bronchiectasis, *Aspergillus* spp. may correlate with poor lung function, and chronic antibiotic treatment may increase the risk of positive fungal isolation.17, 19 Moreover, Aogáin et al. found that *A. fumigatus* and *A. terreus* are dominant in patients in Asia and Europe, respectively, and the latter is associated with exacerbations (Mac Aogain et al., 2018). However, the role of fungal isolates in bronchiectasis is unclear. In most cases, fungi are usually isolated with other pathogens, such as NTM or *P. aeruginosa*, making the determination of their pathogenic significance difficult (Maiz et al., 2018). In the current study, we demonstrated that the risk of respiratory failure were higher in patients with fungus isolates; concomitant *P. aeruginosa* and fungus isolates although the risk of mortality were not significant. Moreover, in patients with concomitant NTM, *P. aeruginosa*, and fungus isolates, the HRs for ventilator use and death were 5.9 and 7.43 times those of the control group. However, the clinical importance of fungus in bronchiectasis was not conclusively demonstrated in our study because of the retrospective nature of our study design and the prevalence of fungus isolates in airway specimen was relatively low in stable bronchiectasis without a suspicion of invasive infection.

Our study had several inherent limitations. First, this was a retrospective study and we could not confirm the bronchiectasis diagnosis because not all our patients were diagnosed using HRCT. The CGRD database is based on real-world clinical practice, and clinicians made the diagnoses of bronchiectasis according to symptoms, history, and radiology reports, which may not be considered a reliable diagnostic tool now. Second, information on some parameters was lacking or incomplete in the CGRD, and hence, we could not derive the BSI. Third, the CGRD could not provide precise pulmonary function, spirometry intervals were inconsistent among patients, and lung function decline could not be calculated. Fourth, the prevalence of fungal isolates in the airway specimen was relatively low in patients with stable bronchiectasis. Therefore, a selection bias may be present and that would diminish the generalizability of the study results. Fifth, the CGRD cohort was mainly obtained from tertiary medical center hospitals, which might mainly treat patients with severe bronchiectasis.

## Conclusion

In summary, patients with non-*CF* bronchiectasis with positive NTM isolates from sputum had frequent exacerbations and were independent predicting factor for mortality. NTM was also associated with respiratory failure while concomitant isolated with *P. aeruginosa*, and fungus. Further prospective, large-scale studies are warranted to validate our results.

## Data Availability Statement

The raw data supporting the conclusions of this article will be made available by the authors, without undue reservation.

## Ethics Statement

The studies involving human participants were reviewed and approved by Institutional Review Board of Chang Gung Memorial Hospital approved this study (IRB number: 201800712B0C502). Written informed consent for participation was not required for this study in accordance with the national legislation and the institutional requirements.

## Author Contributions

C-YL, H-YH, and H-CL: conceptualization. C-YL, M-HH, and Y-FF: investigation. C-YL, Y-LL, S-ML, C-HW, and H-CL: methodology. C-YL, H-YH, Y-TH, and C-HY: data curation. Y-TH and C-HY: validation. C-YL, H-YH, and C-HW: original draft preparation. H-CL: review and editing of manuscript. All authors contributed to the article and approved the submitted version.

## Funding

This study was supported by Chang Gung Memorial Hospital Research Project Grant (CMRPG3F1492, CMRPG3B1323, CMRPG3F1501, CMRPG3F1502, CMRPG3H0931, and CIRPD1D0031; SPMRP-U1–3001).

## Conflict of Interest

The authors declare that the research was conducted in the absence of any commercial or financial relationships that could be construed as a potential conflict of interest.

## Publisher’s Note

All claims expressed in this article are solely those of the authors and do not necessarily represent those of their affiliated organizations, or those of the publisher, the editors and the reviewers. Any product that may be evaluated in this article, or claim that may be made by its manufacturer, is not guaranteed or endorsed by the publisher.

## Footnotes

^1^ http://www.chang-gung.com/en/index.aspx

## Abbreviations

AE: Acute exacerbation
BACI: Bronchiectasis Etiology and Comorbidity Index
BSI: Bronchiectasis Severity Index
*CF*: Cystic fibrosis
CGRD: Chang Gung Research Database
CI: Confidence interval
COPD: Chronic obstructive pulmonary disease
ED: Emergency department
FEV1: Forced expiratory volume in 1 s
HR: Hazard ratio
HRCT: High-resolution computed tomography
NTM: Nontuberculous mycobacteria
