# Supplementary material for: Impacts of Nontuberculous Mycobacteria Isolates in Non-cystic Fibrosis Bronchiectasis: A 16-Year Cohort Study in Taiwan
Source: Front Microbiol. 2022 Apr 18;13:868435. doi: 10.3389/fmicb.2022.868435 (PMC9058169; doi:10.3389/fmicb.2022.868435)
Supplement: Supplementary file 1 [file Data_Sheet_1.docx]

**Table 1S.** Clinical characteristics of study population before and after propensity score matching.

|  | Before matching | | | After matching | | |
| --- | --- | --- | --- | --- | --- | --- |
| Characteristics | Control  (n=10786) | NTM  (n=650) | *p* value | Control  (n=650) | NTM  (n=650) | *p* value |
| Age at diagnosis (mean) | 65.1±14.5 | 64.7±13.5 | 0.513 | 64.4±14.5 | 64.7±13.5 | 0.7215 |
| Gender, male, n (%) | 5072 (47) | 256 (39) | 0.0001 | 256 (39) | 256 (39) | 1.0000 |
| Pulmonary function |  |  |  |  |  |  |
| FEV_1_ ≧ 80 % | 3136 (50) | 188 (40) | 0.3792 | 174 (44) | 188 (40) | 0.2499 |
| 50% ≦ FEV_1_ < 80 % | 2176 (34) | 129 (28) | 0.0192 | 111 (28) | 129 (28) | 0.8607 |
| FEV_1_ < 50% | 1260 (20) | 85 (18) | 0.3611 | 80 (20) | 85 (18) | 0.4365 |
| HRCT |  |  |  |  |  |  |
| Localized, n (%) | 2166 (56) | 188 (49) | 0.0223 | 129 (53) | 188 (49) | 0.3790 |
| Bilateral, n (%) | 972 (25) | 99 (26) | 0.6334 | 62 (26) | 99 (26) | 0.8810 |
| Diffuse, n (%) | 759 (19) | 93 (24) | 0.0199 | 52 (21) | 93 (24) | 0.3757 |
| Etiology |  |  |  |  |  |  |
| Post-infection | 6744 (63) | 419 (64) | 0.3217 | 419 (64) | 419 (64) | 1.0000 |
| Idiopathic | 2053 (19) | 123 (19) | 0.9443 | 114 (18) | 123 (19) | 0.5180 |
| Comorbidity, n (%) |  |  |  |  |  |  |
| Ischemic heart disease | 1291 (12) | 59 (9) | 0.0265 | 74 (11) | 59 (9) | 0.1698 |
| Stroke | 1262 (12) | 55 (8) | 0.012 | 72 (11) | 55 (8) | 0.1123 |
| Diabetes mellitus | 1970 (18) | 83 (13) | 0.0004 | 83 (13) | 83(13) | 1.0000 |
| Liver disorder | 1506 (14) | 94 (14) | 0.7217 | 84 (13) | 94 (14) | 0.4198 |
| Chronic kidney disease | 1013 (9) | 50 (8) | 0.1473 | 47 (7) | 50 (8) | 0.7515 |
| COPD | 4094 (38) | 225 (35) | 0.0879 | 237 (36) | 225 (35) | 0.4868 |
| Asthma | 2874 (27) | 141 (22) | 0.0054 | 141 (22) | 141 (22) | 1.0000 |
| GERD | 838 (8) | 52 (8%) | 0.8312 | 62 (10) | 52 (8) | 0.3268 |
| Hematological malignancy | 2922 (27) | 193 (30) | 0.1479 | 180 (28) | 193 (30) | 0.4254 |
| Connective tissue disease | 1183 (11) | 78 (12%) | 0.4146 | 68 (10) | 78 (12) | 0.3797 |
| Osteoporosis | 1303 (12) | 85 (13) | 0.4499 | 83 (13) | 85 (13) | 0.8687 |
| BACI score | 6.0±5.9 | 5.5±5.6 | 0.0197 | 5.5±5.8 | 5.5±5.6 | 0.8957 |

Abbreviation: NTM: nontuberculous mycobacteria, FEV_1_: forced expiratory volume in one second, HRCT: high-resolution computed tomography, COPD: chronic obstructive pulmonary disease, GERD: gastroesophageal reflux disease, BACI score: The Bronchiectasis Aetiology Comorbidity Index score.

**Table 2S.** NTM species isolated from sputum samples in non-CF bronchiectasis patients.

| NTM species | Single NTM  (n=458) | Multiple NTM  (n=60) | *P. aeruginosa*  (n=89) | Fungus  (n=33) | *P.aeruginosa*  + Fungus  (n=10) |
| --- | --- | --- | --- | --- | --- |
| *M.avium-intracellulare complex* | 234 (45) | 35 (32) | 45 (45) | 14 (33) | 2 (20) |
| *M.fortuitum* | 66 (13) | 21 (19) | 10 (10) | 9 (21) | 2 (20) |
| *M.abscessus* | 58 (11) | 21 (19) | 7 (7) | 4 (9) | 1 (10) |
| *M.gordonae* | 43 (8) | 15 (14) | 12 (12) | 3 (7) | 1 (10) |
| *M.kansasii* | 30 (6) | 10 (9) | 4 (4) | 1 (2) | 0 (0) |
| *M.chelonae* | 27 (5) | 8 (7) | 11 (11) | 4 (9) | 2 (20) |
| Multiple NTM |  | 60 (100) | 10 (10) | 8 (19) | 2 (20) |

Data are presented as n (%).

**Table 3S.** Hazzard ratio of ventilator use according to different NTM species isolates from sputum.

|  | Univariate | | | Multivariate | | |
| --- | --- | --- | --- | --- | --- | --- |
|  | HR | 95% CI | P value | HR | 95% CI | P value |
| *M.avium-intracellulare complex* | 1.01 | 0.70-1.44 | 0.9710 | 1.03 | 0.72-1.47 | 0.8781 |
| *M.fortuitum* | 0.55 | 0.17-1.74 | 0.3082 |  |  |  |
| *M.abscessus* | 0.48 | 0.19-1.17 | 0.1063 | 0.46 | 0.19-1.13 | 0.0899 |
| *M.gordonae* | 1.45 | 0.73-2.88 | 0.2913 |  |  |  |
| *M.kansasii* | 1.09 | 0.58-2.04 | 0.7860 |  |  |  |
| *M.chelonae* | 1.12 | 0.65-1.93 | 0.6948 |  |  |  |
| Multiple NTM | 0.99 | 0.54-1.81 | 0.9640 | 1.07 | 0.58-1.95 | 0.8395 |

*P < 0.05 for comparison between the different NTM species isolates groups and the control group.

NTM, nontuberculous mycobacteria; HR, hazard ratio.

**Table 4S.** Hazzard ratio of mortality according to different NTM species isolates from sputum.

|  | Univariate | | | Multivariate | | |
| --- | --- | --- | --- | --- | --- | --- |
|  | HR | 95% CI | P value | HR | 95% CI | P value |
| *M.avium-intracellulare complex* | 2.63 | 1.24-5.55 | 0.0114 | 2.62 | 1.24-5.56 | 0.0121 |
| *M.fortuitum* | 1.40 | 0.18-10.76 | 0.7467 |  |  |  |
| *M.abscessus* | 1.44 | 0.32-6.42 | 0.6355 |  |  |  |
| *M.gordonae* | 3.37 | 0.95-11.94 | 0.0599 |  |  |  |
| *M.kansasii* | 2.84 | 0.92-8.80 | 0.0707 |  |  |  |
| *M.chelonae* | 1.07 | 0.24-4.79 | 0.9276 |  |  |  |
| Multiple NTM | 5.02 | 2.05-12.29 | 0.0004 | 5.94 | 2.42-14.58 | 0.0001 |

*P < 0.05 for comparison between the different NTM species isolates groups and the control group.

NTM, nontuberculous mycobacteria; HR, hazard ratio.
